# Copeptin Concentrations in Plasma of Healthy Neonates in Relation to Water–Electrolyte Homeostasis in the Early Adaptation Period

**DOI:** 10.3390/children9030443

**Published:** 2022-03-21

**Authors:** Anna Jarosz-Lesz, Aniceta Brzozowska, Iwona Maruniak-Chudek

**Affiliations:** 1 Neonatology Unit, The Guardian Angels Hospital of the Brothers Hospitallers of St. John of God in Katowice, 40-211 Katowice, Poland; jaleszczan@gmail.com; 2 Health Promotion and Obesity Management Unit, Department of Pathophysiology, Faculty of Medicine in Katowice, Medical University of Silesia, 40-752 Katowice, Poland; abrzozowska@sum.edu.pl; 3 Department of Neonatology and Neonatal Intensive Care, Faculty of Medicine in Katowice, Medical University of Silesia, 40-752 Katowice, Poland

**Keywords:** copeptin, newborns, hydration status, electrolyte-water homeostasis

## Abstract

Copeptin (CTproAVP) is a stable by-product of arginine–vasopressin synthesis and reflects its secretion by the pituitary gland, considered as a potential new marker of dehydration. The objective of the study was to investigate CTproAVP measured after the first 48 h of postnatal life in relation to serum effective osmolality, urine osmolality, and vessels filling according to the following variables: delivery mode, postnatal weight loss, fluids administered intravenously to the mother, and fluids given orally to the neonate. A prospective observational study was conducted with 200 healthy term infants (53% male) enrolled. Serum CTproAVP concentrations were measured using the ELISA kit; haematocrit, urine osmolality, serum effective osmolality were assessed after 48 h of life. Sonographic measurements of inferior vena cava (IVC) and aorta (Ao) were performed and IVC/Ao ratios were calculated. No correlations were found between CTproAVP concentrations and both serum effective osmolality and urine osmolality. There was also no association between CTproAVP concentrations and vessel filling represented by IVC/Ao index at 48 h of life.

## 1. Introduction

Water-electrolyte homeostasis in newborn babies in their early adaptation period depends on the course of pregnancy (disturbances in maternal homeostasis), external factors (e.g., intravenous infusions to mothers), and the natural path of the adaptation period, including weight loss, initiation of diuresis, bilirubin concentration, and oral hydration. Newborns are particularly sensitive to the effects of factors disturbing their fluid balance, and disturbances of an initially small degree may lead to serious destabilization of vital functions, and even life threatening conditions, in a relatively short time. Learning about the mechanisms controlling fluid homeostasis, also in the case of moderate disorders of the adaptation period, should help in more precise control of fluid therapy and in better understanding the underlying mechanisms when the newborn is seriously ill (e.g., congenital heart defect) or his adaptation process is substantially disturbed (e.g., perinatal asphyxia). Therefore, the search for an easily available, but precise parameter describing hydration status is justified and not yet completed. Arginine vasopressin (AVP), an essential hormone in body water homeostasis, has been tested to determine hydration status. However, it has never been used in clinical practice due to technical challenges regarding pulsatile release pattern, short plasma half-life, and instability [1]. More recently, human pre-pro-vasopressin split product (copeptin, CTproAVP) secreted in an equimolar ratio to AVP has been introduced as a stable form in the circulation [2]. The marker has been already studied in adults, but to a much lesser extent in children and newborns. CTproAVP concentration has been observed to rise 5-fold [2] with increased osmolality and dehydration, but also in response to psychological stress [3] and exercise (2.5-fold) independent of sodium levels and fluid intake [4]. In response to physical stress, ischemia, and sepsis, copeptin concentrations rise 5- to 100-fold [5,6,7]. Until now, CTproAVP is known as a marker of numerous cardiovascular [8,9,10,11], and renal [9,12,13] conditions. So far, pharmacokinetics and degradation of copeptin are not well examined. It is known from recent studies that in healthy adult volunteers, the elevation in the serum osmolality produced a release of CTproAVP similar to AVP. However, its clearance in the serum was twofold slower than AVP [14] with possible explanations of this mechanism including renal excretion [15] or degradation by tissue-bound proteases. CTproAVP has been studied in cord blood and in neonates, but there are still some questions unanswered, which explain an incorporation of this marker in considerations regarding fluid balance in the neonatal period.

The search for a marker or a method to objectify a patient’s hydration status based on ultrasound evaluation of the diameter of great vessels is particularly known in pediatric and adult emergency medicine. Ultrasound measurements of abdominal inferior vena cava (IVC) diameters have been extensively studied in adults and children to assess intravascular volume status in various clinical conditions and are considered to be a reliable indicator [16,17]. Variations in mean IVC diameter reach up to 30% in children clinically assessed to be severely dehydrated [18] and up to 40% in children undergoing hemodialysis [16]. The bedside ultrasound measurements of the aorta (Ao) and vena cava in their abdominal route allow one to calculate the IVC/Ao ratio, which seems to present lower values in children clinically assessed as dehydrated and increases during the administration of intravenous fluid boluses [19,20,21,22,23].

We performed this prospective study to analyze the serum CTproAVP concentration after the first 48 h of postnatal life according to delivery mode, postnatal body weight loss, and gender in order to better understand the pattern of CTproAVP release in presumably healthy newborns. We compared primary fluid status-related parameters, i.e., serum effective osmolality, urine osmolality, serum sodium, potassium, and glucose concentrations to CTproAVP concentration, and double-checked the fluid status by IVC/Ao measurements ratio. 

## 2. Materials and Methods

A total of 200 term neonates born in 2014 between 1st of March and 30th of November at The Guardian Angels Hospital of the Brothers Hospitallers of St. John of God in Katowice were enrolled after obtaining informed written consent from the parents. The bioethical Committee of the Medical University of Silesia in Katowice approved the study protocol. Infants were considered eligible for enrolment if the birth occurred after completion of 37 weeks (wks) of gestational age (GA). GA was determined by the best estimate from the first day of the mother’s last menstrual period and an early ultrasound examination in the first trimester. All newborns were staying with their mothers in the rooming-in system. Complete patients’ characteristics are presented in Table 1.

Venous blood (up to 500 µL) was collected by peripheral vein puncture on the morning of the 3rd day of life, approximately 48 h (hrs) post-delivery depending on the delivery hour. After centrifugation, serum samples were shortly stored frozen and subsequently transferred to the laboratory for CTproAVP examination. Simultaneously, the blood sample in capillary was submitted for a routine analysis using the Cobas b121 gas analyzer (Roche Diagnostics GmbH Mannheim, Germany). Routine laboratory tests including blood gas analysis, glucose, sodium, potassium and chloride concentrations measurements were performed. Serum effective osmolality (Sosm) was calculated with the following formula: 2 × serum sodium concentration (mM/L) + serum glucose concentration (mM/L)/18.

Serum CTproAVP concentrations were measured using the ELISA kit from USCN (Wuhan, China) with an intra-assay coefficient of variation < 10% and an inter-assay coefficient of variation < 12%. The measurement of freezing point depression determined urine osmolality (Uosm). 

All ultrasound examinations (USex) were performed within the first 48 h of postnatal life with an Acuson X300 ultrasound system (Simens Medical Solutions USA, Inc. Mountain View, CA, USA) using a C8-5 microconvex transducer. In order to exclude possible differences in the measurements when they are performed by different providers, the ultrasound tests were performed only by the same investigator. An USex of abdominal organs was conducted according to standard procedures, to exclude any anomalies. IVC and Ao anteroposterior diameters were measured during the abdominal examination, and the IVC/Ao ratio was calculated. A detailed description of the USex performed in the study was presented in our previous article published in the *Journal of Ultrasound in Medicine* [24] and is cited here: “The infants were placed in the supine position. The transducer was placed over the subxiphoid region and a longitudinal image of each vessel was obtained three times for every measurement. Every two-dimensional (B-mode) image in the form of cine-loop was analyzed frame by frame to find vessel maximal dimension, then anteroposterior diameter of the vessel was measured from inner wall to inner wall. The maximal anteroposterior diameter of the IVC was measured during the expiratory phase of the respiratory cycle about 1 cm beneath the confluence of the hepatic vein, where the IVC walls were parallel to each other. As IVC usually takes the oval shape, the examiner searched for the optimal position to capture best dimension with the probe angling slightly laterally and medially from the sagittal plane during recording cine-loop images. Maximal anteroposterior diameter of Ao was obtained during systole of the cardiac cycle about 1 cm above the mesenteric artery. From three recorded measurements obtained for each vessel, the one with the highest value was selected for further analysis. During the examination, abdominal compression was avoided”.

The medical records data regarding fluids and oxytocin administered to mothers up to 24 h before delivery were obtained. Additionally, the mothers documented their breastfeeding and milk formula amounts (if used) provided to their infants in an individual questionnaire. Any amount of milk formula given to the newborn despite breast feeding was considered as “additional oral fluid”; mothers were encouraged to exclusive breastfeeding, but had unlimited access to milk formula.

Statistical analyses were performed using STATISTICA 10.0 PL for the Windows software package (StatSoft, Tulsa, OK, USA). Values were presented as means with standard deviation or medians with interquartile range, as appropriate. We used the chi-squared test (qualitative variables) and ANOVA to compare groups, followed by Tukey’s test or the U-Man test (quantitative variables). Correlation coefficients were calculated according to Pearson (CTproAVP values were logarithmically transformed for this analysis).

In all statistical tests, the ‘*p*’ values below 0.05 were considered statistically significant.

## 3. Results

### 3.1. General Characteristic of the Study Group

Among 200 term newborns (53% male), 100 were born by vaginal delivery (VD) and 100 by cesarean section (CS). The two groups distinguished based on the mode of delivery were relatively homogeneous (demographic data are presented in Table 1). In 43 cases of deliveries (21.5%), an unexpected stress factor occurred, e.g., the umbilical cord wrapped around the fetal neck, but did not influence clinical condition of the newborn.

The subgroups VD and CS varied considerably regarding oxytocin use, intravenous fluids administered to the mother, and hematocrit values. Neonates born by CS presented higher birth weight (BW) loss on the first day of life and received larger volumes of oral fluids in the first two days. The parameters used to determine hydration status, such as sodium, urine osmolality, and CTproAVP (Table 1), did not present significant differences between the subgroups. IVC/Ao ratio measured at 48 h remained constant regardless of such variables as the delivery mode or gender (Table 1).

### 3.2. CTproAVP 

Comparable CTproAVP values estimated after 48 h of life were observed in all newborns irrespective of the use of oxytocin or fluid transfusion to the mother before or during labor or oral administration of fluids to the neonates (Table 2). 

**Figure 1:**
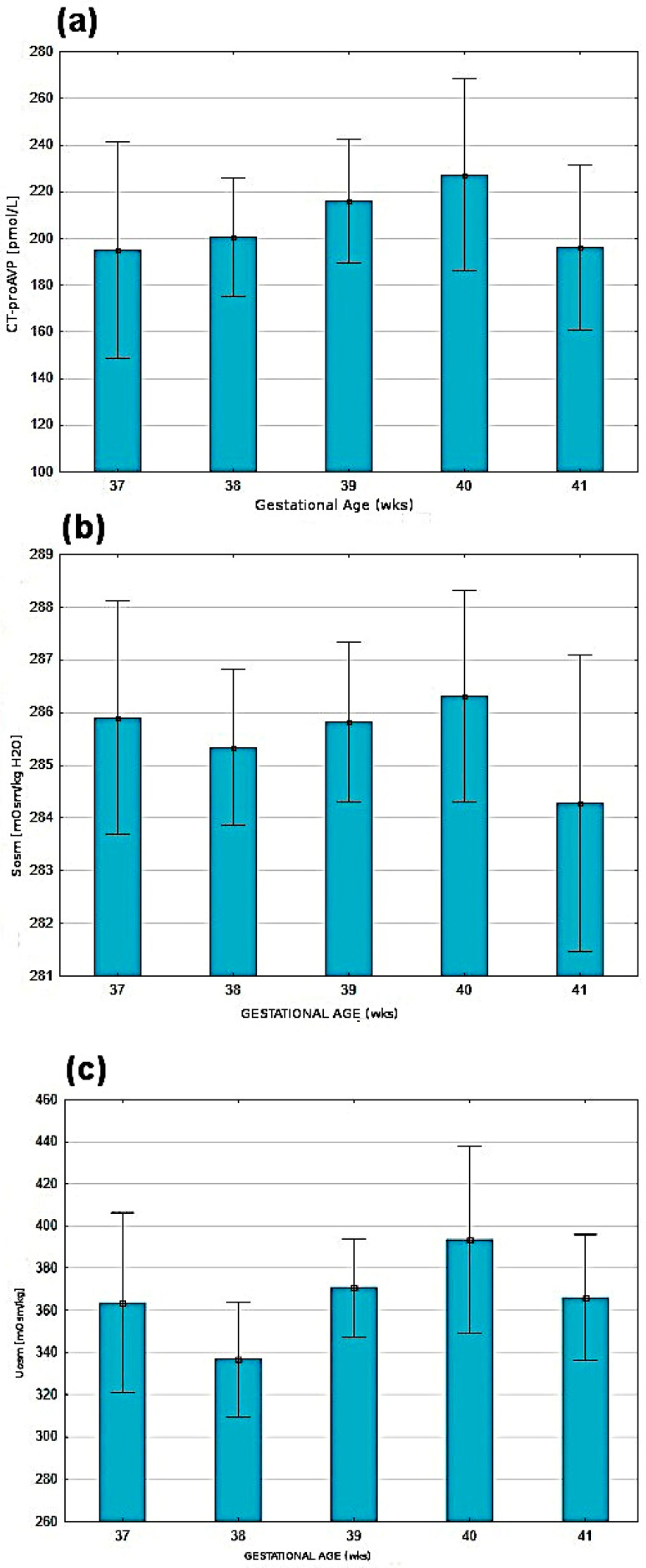
Presentation of selected parameters in relation to gestational age: (**a**) CTproAVP, (**b**) effective serum osmolality (Sosm) and (**c**) urine osmolality (Uosm). The values presented as means *±* SD.

The analysis of demographic factors showed a lack of correlations between gender, BW, five-minute Apgar score (Apgar 5′), and CTproAVP value estimated after the 48th hour of life. There was also no dependency of CTproAVP, Uosm, Sosm in relation to GA (Figure 1). 

There was no correlation found between CTproAVP and effective plasma osmolality or CTproAVP and urine osmolality. Furthermore, no statistically significant correlations were found between the vessels filling represented by IVC diameter and IVC/Ao index and Sosm, Uosm, CTproAVP. We found a similar IVC/Ao index at 48 h regardless of the mode of delivery: 0.62 (CI: 0.33–0.89) (the results were published in our previous publication) [24]. Moreover, there were no correlations between CTproAVP concentration and IVC/Ao index and effective serum osmolality and IVC/Ao index measured after the 48th hour of life. The analyzed variables are presented in Table 3. As expected, statistically significant correlations were found between osmolality measurements in serum and urine, and also when compared to hematocrite and sodium values (Table 3). 

## 4. Discussion

We designed the study to investigate the potential for AVP impact on changes in water status in healthy term neonates. The VD and CS groups were highly homogenous in terms of GA, birth weight, and the absence of substantial stress factors that might have affected the measurements. In both groups, serum effective osmolality after the 48th hour of life was almost within the physiological range, 273–297 mOsm/kg H_2_O, which agrees with the results of other studies [25]. The whole sample osmolality of urine collected before the 48th hour of life was 365 *±* 105 mOsm/kg H_2_O. It is a well-known fact that in neonates, urine secretion increases after the first 12 h of life while renal concentration capacity is reduced [26]. This phenomenon may be related to limited glomerular filtration, the diminished function of the nephron loop, and the relatively low sensitivity of the renal distal and collecting tubules to the AVP, which might result from local renal production of the prostaglandins inhibiting AVP [27]. The results of other studies regarding the impact of delivery mode and fluids given intravenously to the mother before or during the delivery on hydration status in neonates are inconclusive [28,29,30,31].

Intravenous infusion of fluids to the mother prior to delivery may increase volemia in the fetus or neonate, resulting in increased diuresis in the first days of postnatal life and subsequent excessive weight loss during the first two days after birth [28,29,31]. Studies regarding the influence of breastfeeding versus formula feeding on neonates’ voiding gave inconsistent results [32,33,34]. Our research did not find any relationship between urine osmolality and fluids administered intravenously to the mother. Fluids administered orally to the neonate in addition to breastfeeding also did not influence urine osmolality. Urine osmolality was slightly (statistically insignificantly) lower in the group of neonates born by CS. A tendency to higher values of urine osmolality in the group of neonates born by VD may be due to delayed first voiding in those neonates, as was demonstrated in other studies [35]. This tendency, while not reaching statistical significance, might be associated with a small sample size.

Parturition and the first few days of extrauterine life are considered among the most stressful events in human life, associated with activating the neurohormonal system and releasing vast amounts of neurohormones [36,37,38,39]. Before the delivery, CTproAVP concentration in fetal blood depends on fetal distress [40,41] and increases during contractions induced in oxytocin challenge test- before elective cesarean delivery [42]. CTproAVP concentration rises during labor, probably due to fetal production [43]; however, the possibility of placental transfer during labor is also considered [44]. Infants born by noncomplicated VD present lower CTproAVP concentrations in umbilical cord blood than infants born by instrumental VD or urgent CS but higher than those born by primary CS [41,45]. CTproAVP concentrations increase as a function of labor duration and the duration of the second stage of labor [46]. In case of birth asphyxia, CTproAVP concentration is significantly higher, correlating with umbilical artery pH and base excess [36,38,39,40,46]. The same relationship was observed for low-risk term deliveries complicated by meconium-stained amniotic fluid [44]. This phenomenon accompanies the negative correlation between cord blood CTproAVP concentration and the magnitude of the noxious-evoked brain activity observed in newborns a few hours after birth [47]. CTproAVP serum concentration progressively declines after delivery in the first 48–72 h of life [48]. Maximum weight loss (typically on the 2nd–5th day of life) correlates directly with CTproAVP concentrations in venous plasma drawn on the 3rd day of life [38]. All listed factors raise a question if and to what extend CTproAVP can be considered a marker of hydration status in the early adaptive period.

We performed the measurements of CTproAVP concentration on the third day of postnatal life. The mean serum concentration of CT-proAVP was 208.7 *±* 106.1 pmol/L. It should be emphasized that we did not measure CTproAVP concentration in umbilical blood. Due to that fact, we cannot discuss the outcomes presented in other papers investigating the issue in relation to delivery mode. All participants were born in good clinical condition, and even though some unexpected stress factors occurred in the perinatal period, we did not notice any particular increase in this marker. With respect to CTproAVP values, the differences between newborns classified according to delivery mode were noticed, yet they were statistically insignificant. Our findings agree with the results presented in other studies, which showed that the correlation vanished by the third day of life [36,38,39,49].

Similarly, slight differences in CTproAVP concentrations between male and female neonates on the third day of their life may reflect the sexual disparity of CTproAVP concentration vanishing at around 72 h of life, as was observed in the other studies [49]. Our study did not find significant correlations between birth weight values and CTproAVP concentrations, which is consistent with findings by Wellman et al. [38], but in opposition to other studies [36,49]. We observed an increasing trend of CTproAVP in the subsequent GA ranges, which was shown more clearly by Benzing et al. [36]. It is well known that BW loss is observed mainly in the first three days of neonatal life. Some researchers [35] found a correlation between higher CTproAVP concentrations in umbilical blood and a less rapid decrease in BW as well as delayed first urination. Regarding delivery mode, the findings of our study show substantial variations between the groups in terms of BW loss percentage on the first day of postnatal life, with newborns born by CS presenting higher values. We did not find a statistically significant correlation between BW loss and CTproAVP concentration on the third day of life, which is inconsistent with other studies in which such a correlation was shown [38,49]. We investigated potential correlations between CTproAVP concentration and hematocrit and several biochemical parameters (pH, bilirubin, glucose); however, we failed to establish any significant correlations similarly as presented by Wellman et al. [38]. The discrepancy between our findings and those presented in the study by Benzing et al. [36] might be due to differences in study populations, as our study group consisted only of healthy term neonates. The results of our study showed that during the period of early physiological adaptation to extrauterine life, CT-proAVP concentration (therefore, AVP) on the third day of life is not associated with previously conducted interventions, such as fluid infusions given parenterally to mothers 24 h before delivery, oxytocin administration to women in labor, or fluids provided orally to neonates in addition to breastfeeding. Therefore, it may be assumed that CT-proAVP concentration on the third day of postnatal life reflects the actual AVP concentration in the neonate’s organism, in normal ranges, undisturbed by minor medical interventions. 

In the medical literature, we found several publications on the relationship between the diameter of large vessels (Ao, IVC) and the IVC/Ao ratio and the stage of hydration. [17,19,20,21] These descriptions indicated the possibility of using the abovementioned measured and calculated parameters at the bedside to assess the filling of the vascular bed and the effects of the fluid therapy applied. The neonatal population as a group is different from the general pediatric population and varies considerably, especially when the body weight and adaptation changes in perinatal period are taken into account. There is only one study investigating vessels filling in neonates by measurement of IVC, Ao, and IVC/Ao ratio [50]; however, in this research, the measurements were performed on various days of neonates’ lives. In our study, to all participants, the procedures of delayed cord clamping or cord milking were applied. Therefore, we assume the neonates were not affected by blood volume depletion due to delivery. Moreover, all study participants were fed orally (breastfed) and did not receive any fluids intravenously. Consequently, the results of our study regarding vascular measurements of IVC, Ao, and IVC/Ao ratio may reflect the physiologic adaptation of the circulatory system and a sort of neonatal equilibrium. Our findings agree with previous studies on VLBV preterm neonates, which demonstrated that postnatal weight loss of 7.8% of BW resulted in a decrease in interstitial volume without changes in blood or plasma volumes [51].

We did not find any significant correlations among vascular filling and hematocrit, biochemical parameters, serum effective osmolality, and urine osmolality. We also did not find any significant correlations between vascular filling and CT-proAVP concentration. 

A few explanations of these phenomena are possible: 

1. In physiological conditions (without additional stress factors), vascular filing is sufficient. Therefore, there is no trigger for receptors in vessel walls to transmit signals of hypovolemia to the central nervous system. As a result, the secretion of AVP and CT-proAVP is not increased even in the events of initial neonatal body weight loss and variations in total body water (TBW).

2. In the case of the lower sensitivity of the renal distal and collecting tubules to the AVP, the regulation of volemia is controlled by a different hormone or natriuretic peptide, hence the lack of correlation between the vessel diameters and CT-proAVP (AVP) concentration.

3. The range of observed changes in the vascular filling was too narrow to produce significant differences in the secretion of CTproAVP (AVP). 

## 5. Conclusions

The results of our study suggest that in term neonates, the impact of perinatal factors, such as oxytocin use, fluids administered intravenously to the mother, and the mode of delivery on CTproAVP concentrations vanish by the third day of postnatal life (based on the lack of relationship between them and the marker’s value). It can be assumed that CT-proAVP concentration marked after the first 48 h of life reflects the actual AVP concentration in the neonate’s organism. Vascular filling evaluation in healthy neonates based on the diameter of Ao and IVC, and IVC/Ao ratio after the first 48 h of life is not related to CTproAVP concentration, and it appears that other mechanisms exist that are responsible for adequate vascular filling despite neonatal body weight loss. Future research on this topic is needed.

## Figures and Tables

**Table 1:** Characteristics of all enrolled patients, divided into two subgroups dependent of the mode of delivery (VG—vaginal delivery; CS—cesarean section, Sosm—effective serum osmolality, Uosm—urine osmolality). Mean and ± SD are presented.

Parameter/Group	All (N = 200)	VG (N = 100)	CS (N = 100)
Gender (M/F) [N]	107/93	57/43	50/50
Gestational age [wks]	39 ± 1	40 ± 1	39 ± 1
Birth weight [g]	3385 ± 406	3421 ± 356	3349 ± 450
Apgar 5′ [pts]	9.9 ± 0.4	9.9 ± 0.4	9.9 ± 0.3
Birth weight loss in 48 h [%]	6.6 ± 1.7	6.2 ± 1.7	6.9 ± 1.6
IVC diameter at 48 h [mm]	4.1 ± 0.5	4.1 ± 0.6	4.0 ± 0.5
IVC/Ao at 48 h	0.62 ± 0.08	0.62 ± 0.08	0.61 ± 0.08
Hematocrit at 48 h [%]	50.4 ± 5.6	52.6 ± 4.5	48.3 ± 5.7
Blood pH at 48 h	7.38 ± 0.03	7.38 ± 0.03	7.37 ± 0.03
Serum bilirubin at 48 h [mg/dL]	8.1 ± 2.7	8.5 ± 3.0	7.8 ± 2.3
Serum sodium at 48 h [mmol/L]	140.8 ± 3.1	140.6 ± 3.3	141.0 ± 2.9
Serum potassium at 48 h [mmol/l]	4.36 ± 0.44	4.32 ± 0.41	4.40 ± 0.47
Serum glucose at 48 h [mg/dL]	66.9 ± 10.4	67.2 ± 10.3	66.5 ± 10.6
Sosm—48 h [mOsm/kgL]	285 ± 6	285 ± 7	286 ± 6
Uosm [mOsm/kg]	364.9 *±* 105	371 *±* 34	359 *±* 27
CTproAVP at 48 h females [pmol/mL]	205 ± 108	224 ± 112	186 ± 104
CTproAVP at 48 h males [pmol/mL]	213 ± 102	213 ± 116	214 ± 89

M—males, F—females.

**Table 2:** Table: urine osmolality (Uosm), plasma effective osmolality (Sosm), CTproAVP in relation to oxytocin and fluids given intravenously to mothers or fluids given orally to neonates regardless of breastfeeding. Values presented as means *±* SD.

Parameter	Action	N	CTproAVP [pmol/L]		Uosm [mOsm/kg H_2_O]		Sosm [mOsm/kg H_2_O]	
Oxitocin given to mother	No	150	214 ± 121	*p* = 0.36	353 ± 88	*p* = 0.38	286 ± 6	*p* = 0.7
	Yes	50	207 ± 101		369 ± 11		285 ± 7	
Fluid given to mother IV	No	132	206 ± 100	*p* = 0.33	360 ± 92	*p* = 0.91	286 ± 6	*p* = 0.66
	Yes	68	213 ± 117		375 ± 12		285 ± 7	
Fluid given to neonate 0–24 HOL ^1^	No	116	215 ± 119	*p* = 0.56	361 ± 96	*p* = 0.83	285 ± 6	*p* = 0.51
	Yes	84	205 ± 96		370 ± 11		286 ± 7	
Fluid given to neonate 25–48 HOL	No	100	206 ± 99	*p* = 0.73	376 ± 100	*p* = 0.89	286 ± 7	*p* = 0.73
	Yes	100	212 ± 113		362 ± 11		286 ± 6	

^1^ HOL—hours of life.

**Table 3:** Univariate correlations (according to Spearmann) between CTproAVP, effective serum osmolality (Sosm), urine osmolality (Uosm), and biochemical parameters and IVC/Ao ratio.

Parameter/Groups	Sosm		Uosm		CTproAVP	
	r	*p*	r	*p*	r	*p*
Bilirubin	0.08	0.274	0.05	0.502	0.05	0.531
Hematocrit	−0.28	0.001	0.08	0.001	−0.11	0.109
pH	0.01	0.924	0.11	0.112	0.06	0.413
Na+	1.00	0.001	0.21	0.003	0.14	0.052
K+	−0.14	0.049	−0.09	0.028	0.03	0.703
Glucose	−0.10	0.177	−0.09	0.185	−0.11	0.168
Uosm	0.21	0.004	X	X	−0.05	0.072
Sosm	X	X	0.21	0.010	0.13	0.068
IVC/Ao 48 HOL ^1^	0.00	0.520	0.05	0.423	−0.01	0.089

^1^ HOL—hour of life.

## Data Availability

The data presented in this study are available on request from the corresponding author. The data are not publicly available due to local regulations.
